# Bond Strength of Self-Adhesive Flowable Composites and Glass Ionomer Cements to Primary Teeth: A Systematic Review and Meta-Analysis of In Vitro Studies

**DOI:** 10.3390/ma14216694

**Published:** 2021-11-06

**Authors:** Flavia Iaculli, Alessandro Salucci, Gianni Di Giorgio, Valeria Luzzi, Gaetano Ierardo, Antonella Polimeni, Maurizio Bossù

**Affiliations:** 1 Department of Neuroscience and Reproductive and Odontostomatological Sciences, University of Naples Federico II, 80131 Naples, Italy; flavia.iaculli@unina.it; 2 Department of Oral and Maxillofacial Science, “Sapienza” University of Rome, 00161 Rome, Italy; alessandro.salucci@uniroma1.it (A.S.); valeria.luzzi@uniroma1.it (V.L.); gaetano.ierardo@uniroma1.it (G.I.); antonella.polimeni@uniroma1.it (A.P.); maurizio.bossu@uniroma1.it (M.B.)

**Keywords:** bond strength, dental restorations, glass ionomer cements, primary teeth, self-adhesive flowable composites

## Abstract

***Background***: Conventional composites are largely used in pediatric restorative dentistry and demonstrate successful clinical outcomes. However, the need for simplification of operative steps in young or uncooperative children demands reliable alternatives. Therefore, the aim of the present systematic review and meta-analysis was to evaluate the in vitro bond strength of glass ionomer cements (GICs) and self-adhesive flowable composites (SFCs) on deciduous teeth. ***Methods***: A comprehensive literature search according to the PRISMA checklist was manually and electronically performed by two independent reviewers through the following databases: MEDLINE/PubMed, Google Scholar, Scopus, and Embase, to include in vitro studies comparing GICs and SFCs bond strength values of restorations on primary teeth. In addition, three groups of meta-analyses were conducted using random-effects models. ***Results***: Three articles meeting the inclusion criteria were selected and subjected to both qualitative and quantitative assessment. No statistically significant difference was found between SFC versus GIC; however, both groups significantly differed with conventional flowable composites (CFs). ***Conclusions***: Despite the absence of significant difference in bond strength values, SFCs may be considered a valid alternative to GICs in the restoration of deciduous teeth, although CFs proved better in vitro performances.

## 1. Introduction

The introduction of composite resins in 1962 [1] revolutionized restorative dentistry, allowing more conservative, predictable, and highly aesthetic dental reconstructions [2,3]. Retention and stability of composite resins on dental tissue are provided by adhesive systems through the creation of a micromechanical bond [4,5]. Over the years, adhesive systems have improved, increasing bonding properties and enhancing the interaction between the resinous functional monomers and the mineral constituents of hydroxyapatite by the formation of chemical bonds [6,7]. The latest generations of adhesives allow effective, long-lasting bonds and a simplification of the operative steps to be obtained [8,9]. However, the quality and duration of the adhesive bond [10,11] strictly depend on the isolation of the operating field from the oral environment with the aim of also preventing dental contamination.

Glass ionomer cements (GICs) consist of a calcium fluoroaluminosilicate powder and an aqueous solution of polyacrylic acid, which are combined through an acid–base reaction [12]. They are mainly characterized by high biocompatibility [13,14], thermal expansion coefficient comparable to dental tissue [15], chemical adhesion to tooth surfaces without any pre-treatment [12], and in situ release of fluoride [16]. GICs are widely used in pediatric dentistry as they result in a simplification of clinical procedures and can be applied in a single mass, significantly reducing the chair time [17,18]. Furthermore, thanks to their hydrophilic properties, GICs have demonstrated a good tolerance to wet substrates unlike composite resins [19]. This ability is particularly advantageous in those clinical situations in which isolation of the operating field may be difficult. In addition, acting as a reservoir of fluoride, they are widely used in the control and prevention of caries in young patients [20,21,22,23,24]. Despite having considerable advantages, conventional GICs are characterized by insufficient physical and mechanical properties as well as aesthetic limitations [25,26,27], restricting their use in the restorative field.

Recently, self-adhesive flowable composites (SFCs) have been introduced to reduce operating times and sensitivity related to clinical procedures [28]. SFCs have a chemical composition similar to traditional composites with the addition of acid functional monomers (such as 10-methacryloyloxydecyl dihydrogen phosphate (10-MDP) or glycerol phosphate dimethacrylate (GPDM)), which allow conditioning of enamel and dentin and formation of chemical bonds with inorganic components of the tooth structure [29]. Moreover, the presence of resinous monomers leads to the establishment of a micromechanical retention [30,31]. Nevertheless, these materials demonstrated a lower bond strength than conventional composite resins using both self-etch or etch and rinse adhesive systems [32,33,34,35]. Since SFCs do not require pre-treatment of dental structure and simplify the restorative procedures [34], they have recently been proposed for conservative pediatric treatments, mainly in cases of young or uncooperative children in which rubber dam isolation is quite difficult, and might be considered as a reliable alternative to GICs. However, further studies are needed to assess the bonding properties of different restorative materials on primary teeth.

Therefore, the aim of the present study was to systematically review the scientific literature to evaluate in vitro studies comparing bond strength of GICs and SFCs on primary teeth. The null hypothesis is that there is no difference in bond strength values between GICs and SFCs.

## 2. Materials and Methods

The present systematic review was performed in accordance with the guidelines of the established Preferred Reporting Items for Systematic Reviews and Meta-Analyses (PRISMA) statement [36]. The protocol was registered on PROSPERO (CRD4202126163). The review question, “Is the bond strength of self-adhesive flowable composites comparable or even better than glass ionomer cements to primary teeth?”, was formulated using the PICOS (Population; Intervention; Comparison; Outcome; Study Design) framework as follows:

Population: Primary teeth.

Intervention: Self-adhesive flowable composites.

Comparison: Glass ionomer cements.

Outcome: Bond strength.

Study design: Comparative in vitro studies.

### 2.1. Search Strategy

The literature search was performed until 1 June 2021 by two independent reviewers (F.I., A.S.) and was based on the following electronic databases: MEDLINE/PubMed, Google Scholar, Scopus, Embase. Free text terms or, when possible, MeSH keywords were used alone or combined with the Boolean operators ‘AND’ and ‘OR’ as follows: Deciduous Tooth, Primary Tooth, Primary Dentition, Deciduous Dentition, Self-Adhesive Composite, Self-Adhering Composite, Self-Adherent Composite, Glass Ionomer Cement, Bond Strength. In addition, a search was also conducted on relevant journals on the topic such as *Journal of Adhesive Dentistry, International Journal of Paediatric Dentistry, European Journal of Paediatric Dentistry, Journal of Esthetic and Restorative Dentistry, Pediatric Dentistry* with the objective of evaluating all available in vitro studies; moreover, reference lists of the identified studies underwent hand search.

### 2.2. Eligibility Criteria

Studies were selected according to the following criteria.

Inclusion Criteria:

- Articles published until June 2021 in peer-reviewed Journal considering unlimited publication years;
- English language;
- In vitro comparative studies;
- Studies that included primary teeth restored with self-adhesive flowable composites and glass ionomer cements evaluating bond strength.

Exclusion Criteria:

- In vivo studies, animal studies, reviews, case reports, case series;
- Studies on permanent teeth;
- Studies without comparison between self-adhesive flowable composites and glass ionomer cements in terms of bond strength.

### 2.3. Screening and Selection of Studies

The resulting papers were screened by two independent reviewers (F.I., A.S.) importing all studies on a commercially available software program (MENDELEY, Mendeley Ltd., London, UK) able to remove duplicates. Then, studies underwent assessment of title and abstract according to the eligibility criteria. Papers that seemed to meet the inclusion criteria were selected for full-text analysis. Only articles that fulfilled the eligibility criteria were included. Exclusion reasons were provided. Controversies between the two authors (F.I. and A.S.) during studies selection were discussed with an additional expert (M.B.).

Agreement level among the two authors was assessed by means of the Cohen’s kappa coefficient (k).

### 2.4. Data Extraction

Data were extracted and recorded using a standardized extraction form built in Microsoft Excel 2020 (Microsoft Corporation, Redmond, WA, USA). Specifically, the following details were collected: authors, year, journal, title, study design, aim of the study, type of used self-adhesive composites/glass ionomer cements, groups distribution, intervention, evaluated parameters, reported outcomes, assessment of risk of bias.

### 2.5. Quality Assessment

Risk of bias evaluation was performed according to a very recent systematic review and meta-analysis on a similar topic [34]. Specifically, the following parameters were assessed in each included article: random sequence generation, sample-size calculation, presence of a clearly defined control group, blinding of the operator or examiner, and other bias such as absence of caries and cracks on enrolled teeth, use of materials according to the manufacturers’ instructions, thermocycling/aging before bond strength test, and type of applied loading.

If the parameter was described in each study, it was considered to be of low risk of bias. Conversely, if the required information could not be retrieved, the paper was considered high risk. Controversies between the two authors (F.I. and A.S.) were discussed to reach a univocal agreement.

### 2.6. Data Analysis

A meta-analysis was conducted using Review Manager 5 (RevMan current version: 5.3.5). Mean differences were combined for continuous data, using either fixed-effects models or, in the presence of heterogeneity among studies, random-effects models. Three groups of meta-analyses were performed based on the bond strength of three different cement in the primary teeth:

- Self-adhesive flowable composite versus glass ionomer cement;
- Conventional flowable composite versus glass ionomer cement;
- Conventional flowable composite versus self-adhesive flowable composite.

## 3. Results

**Figure 1:**
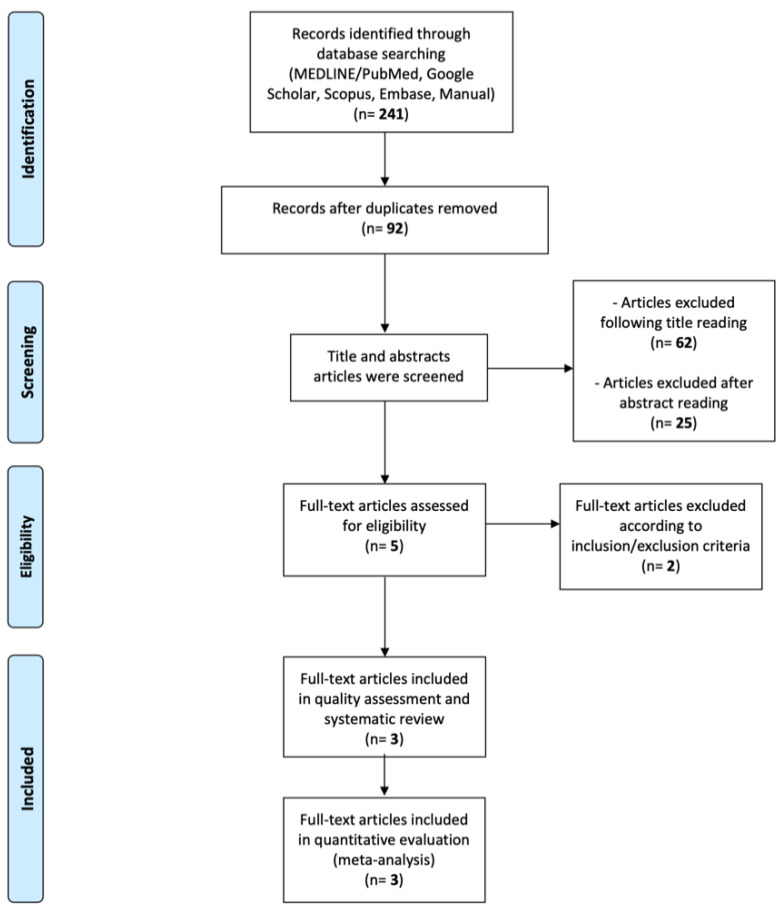
PRISMA flow-chart.

A total of 241 relevant articles were identified through a search of electronic databases and a hand search. After duplicates removal, 92 articles underwent title assessment and a total of 30 papers were further evaluated for abstract reading. Finally, five potential full-text articles were retrieved and assessed. Two articles were excluded since they reported on permanent teeth [33,37]. Three in vitro comparative studies [38,39,40] were included in the present systematic review and in the quality assessment; the same studies underwent quantity evaluation (meta-analysis) (Figure 1).

### 3.1. Characteristics of the Included Studies

All evaluated studies included at least three experimental groups, comparing the bond strength on primary teeth of SFC, GIC, and conventional flowable composite (CF). All specimens restored with self-adhesive composites received no surface pre-treatment; conversely, adhesion procedures were performed in samples restored with flowable composite using both one-step [38,39] or two-step approaches [38,40]. Experimental groups restored with GIC [38,39] or resin-modified glass ionomer cements (RMGIC) [38,40] underwent surface pre-treatment with polyacrylic acid in two out three studies [38,39] and no surface treatment in one study [40].

Restorations made by CF resulted in a significantly higher shear bond strength than other groups, in all included studies. Comparing SFC and GIC, controversial results were shown. Specifically, Poorzandpoush et al. [40] and Scaminaci Russo et al. [39] reported higher shear bond strength for SFC, statistically significant in the latter. Conversely, Pacifici et al. [38] demonstrated better bond strength values in cases of both RMGIC and GIC than SFC, although the differences were not statistically significant.

Concerning the mode of failure, cohesive fractures were reported in specimens restored with CF [38,39,40]. On the contrary, groups restored by GIC/RMGIC [38,40] reported mostly adhesive failures or mixed ones [39]. Regarding SFC, Pacifici et al. [38] reported adhesive failures, whereas the other two included studies [39,40] demonstrated cohesive failures comparable to those of conventional flow composites groups.

Characteristics of the included studies are summarized in Table 1.

### 3.2. Assessment of Risk of Bias

**Figure 2:**
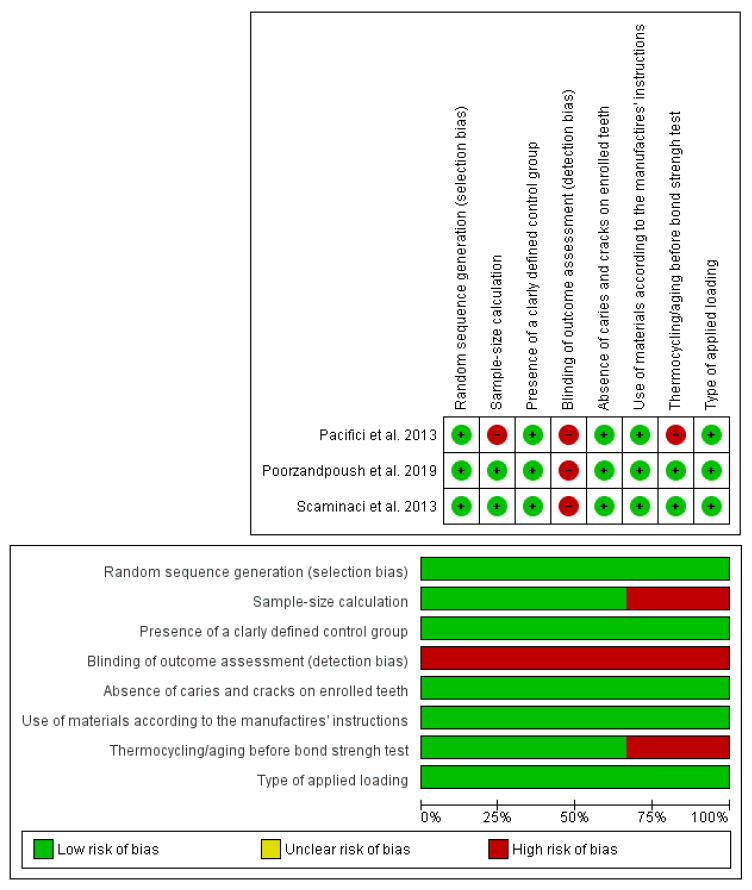
Quality assessment and risk of bias.

The bias risks are reported in Figure 2. The assessment was conducted using the checklist of Cochrane Collaboration’s tool for evaluating the risk of bias [41] excluding domains 3, 5, and 6 and adding personalized domains 5, 6, 7, and 8. The shortcomings mostly concerned the domain “Blinding of outcome assessment”, which was not satisfied in all studies [38,39,40]. Moreover, the study conducted by Pacifici et al. [38] demonstrated a high risk of bias related to the sample size calculation and thermocycling/aging before bond strength test. Cohen’s kappa value for global inter-reviewer agreement was perfect, being 100% in agreement.

### 3.3. Results of the Meta-Analyses

**Figure 3:**
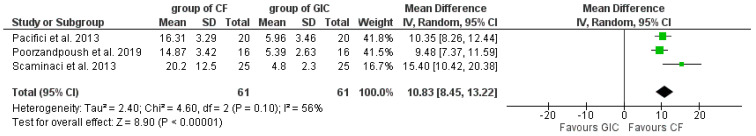
Forest plot of the comparison of bond strength between conventional flowable composite (CF) and glass-ionomer cement (GIC).

**Figure 4:**
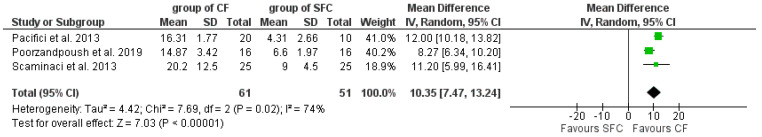
Forest plot of the comparison of bond strength between conventional flowable composite (CF) and self-adhesive. flowable composite (SFC).

**Figure 5:**
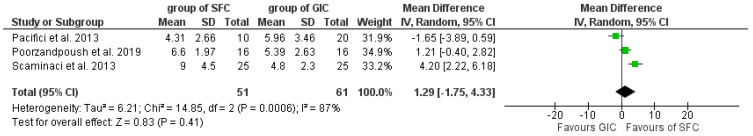
Forest plot of the comparison of bond strength between self-adhesive flowable composite (SFC) and glass-ionomer cement (GIC).

The meta-analysis showed significant difference in the bond strength between CF versus GIC (Mean Difference (MD) 10.83; 95% CI 8.45 to 13.22, *p* < 0.00001, heterogeneity: Tau2 = 2.40; Chi2 = 4.60, df = 2 (*p* = 0.10); I2 = 56%) (Figure 3), and between CF versus SFC (Mean Difference (MD) 10.35; 95% CI 7.47 to 13.24, *p* < 0.00001, heterogeneity: Tau2 = 4.42; Chi2 = 7.69, df = 2 (*p* = 0.02); I2 = 74%) (Figure 4). No statistically significant difference was found between SFC versus GIC (Mean Difference (MD) 1.29; 95% CI −1.75 to 4.33, *p* = 0.41, heterogeneity: Tau2 = 6.21; Chi2 = 14.85, df = 2 (*p* = 0.0006); I2 = 87%) (Figure 5).

## 4. Discussion

Bond strength is an essential prerequisite for the sealing and long-term success of dental restorations [7,42]. This aspect should be also taken into account in primary teeth due to a lesser mineralization and difference in enamel microstructures of deciduous enamel than permanent ones [43,44], which affects the bonding [45].

The present systematic review and meta-analysis showed that there was no statistically significant difference in the bond strength of GICs and SFCs on deciduous teeth, accepting the null hypothesis. On the other hand, CFs performed significantly better than GICs and SFCs, respectively. These results are in agreement with a very recent review [34] on the same topic that demonstrated lower bond strength values of SFCs than conventional composite resins on both permanent and deciduous teeth. The difference in bond strength is not unexpected due to the well-known weak bonding of both GICs and SFCs to dental tissues [30,46,47]. Indeed, GICs showed an adhesion to tooth surfaces by ionic bonds between the carboxylated functional groups of the cement and the calcium ions of hydroxyapatite [48]. Although this bond is also strengthened by a micromechanical retention, due to interlocking of cement tags in the dentinal structure, its entity remains low [46]. In addition to this, the bond is greater for enamel than dentin, suggesting that it mainly occurs with the mineralized component of the tooth [46]. This aspect is again critical in primary teeth, which demonstrated a lesser degree of mineralization than permanent dental elements [43]. In the same way, SFCs demonstrated a high viscosity that negatively interfered with wettability and etching, decreasing the adhesion properties [49,50]. Conversely, GICs and SFCs have been proposed as alternatives to conventional composites in pediatric restorative dentistry due to simplification of operative steps and more tolerance to the absence of field isolation [51,52]. Particularly, SFCs avoid etching with phosphoric acid, which as a strong acid, is more invasive for the thin dentin thickness of deciduous teeth and may limit the efficacy of bonding [40,53], even resulting in post-operative sensitivity.

All the studies included in the present systematic review considered SFCs to be slightly better than GICs in terms of bond strength values, although this difference did not show statistical significance according to the performed meta-analysis. This is due to the limited number of included studies and total number of specimens. However, it could be speculated that this trend in favor of SFCs would be statistically significant with an increased sample size. Although the performed meta-analysis presented low statistical power due to the paucity of included studies as well as limited sample size, it provided a preliminary overview on the topic, especially considering that studies on primary teeth are limited. Indeed, studies evaluating bond strength values of restoration in primary teeth, and more in general, well-conducted randomized clinical trials including primary teeth with adequate sample size and long follow-up period, are limited and should be prospectively performed.

Furthermore, the main limitation of the present study was the inclusion of only three in vitro studies that showed heterogeneity mainly in technical procedures and performance of the SBS test. Inclusion of in vitro studies better standardizes the outcomes’ assessment; however, clinical protocols are necessary to avoid the dissimilarities between the in vitro settings and in vivo oral conditions that could interfere with the results. Specifically, to overcome this limit, the risk of bias assessment was modified to include special domains evaluating the samples features, aging and type of applied loading, to better simulate the clinical condition.

Despite the absence of significant difference in bond strength values, SFCs may be considered as a valid alternative to GICs in the restoration of primary teeth, although bonding stability over time should be improved and evaluated in depth in further studies.

## 5. Conclusions

Within the limitation of the present systematic review and meta-analysis, it can be concluded that self-adhesive flowable composites may be used as an alternative to glass ionomer cements in pediatric restorative dentistry, even though conventional flowable composites show better values of in vitro shear bond strength.

## Figures and Tables

**Table 1:** Summary of included studies.

	Pacifici et al. 2013 [38]	Scaminaci Russo et al. 2013 [39]	Poorzandpoush et al. 2019 [40]
Aim of the study	To evaluate SBS to dentin of primary molars of SFC, GIC, RMGIC, and CF in combination with two different adhesive systems.	To compare µSBS to human primary dentin of SFC, self-etch adhesive + CF, and GIC.	To compare SBS of SFC, CF, and RMGIC to primary dentin.
Type of SFC	Vertise Flow (Kerr, Orange, CA, USA).	Vertise Flow (Kerr, Orange, CA, USA).	Vertise Flow (Kerr, Orange, CA, USA).
Type of GIC or RMGIC	Fuji II LC Capsule (GC Corp., Tokyo, Japan); Fuji IX GP Fast Capsule (GC Corp., Tokyo, Japan).	Ketac Fil (EMS, Milano, Italy)	Ionolux^®^ (VOCO Dental, GmbH, Cuxhaven, Germany).
Group Distribution and Intervention	G1 (*n* = 10): Total-etch adhesion + CF; G2 (*n* = 10): Self-etch adhesion + CF; G3 (*n* = 10): Polyacrylic Acid + GIC; G4 (*n* = 10): Polyacrylic Acid + RMGIC; G5 (*n* = 10): SFC (no surface pre-treatment). *SBS test*: After restoration, samples were positioned in a universal testing machine. Load was parallel to the bonded interface at a crosshead speed of 1 mm/min until failure.	G1 (*n* = 25): SFC (no surface pre-treatment); G2 (*n* = 25): Self-etch adhesion + CF; G3 (*n* = 25): polyacrylic acid+ GIC. *SBS test*: After restoration, samples were stored in water in a light-proof container at 37 °C for 24 h and then thermocycled for 1500 cycles between 5 and 55 °C. Then, specimens were positioned in a universal testing machine. Load was applied to the resin/dentin interface at a crosshead speed of 1 mm/min until failure.	G1 (*n* = 16): GIC (no surface pre-treatment); G2 (*n* = 16): Total-etch adhesion + CF; G3 (*n* = 16): SFC (no surface pre-treatment). *SBS test*: After restorations, samples were thermocycled for 1000 cycles between 5 and 55 °C. Using a universal testing machine, load was applied perpendicular to the tooth-restoration interface at a crosshead speed of 1 mm/min and until bond failure.
Evaluated outcomes	SBS (Mpa) + mode of failure (adhesive; cohesive or mixed).	µSBS (Mpa) + mode of failure (adhesive; cohesive dentin failure; cohesive build-up failure; mixed with 1 and 2 and mixed failure with 1 and 3.	SBS (Mpa) + mode of failure (adhesive; cohesive or mixed).
Results	*SBS*: G1 > G2 > G4 > G3 > G5 G1 showed significantly higher SBS values than all the other tested materials. SBS achieved by G5 was statistically comparable to G3 and G4. *Mode of failure*: Cohesive failures within dentin only in G1 and G2. Adhesive failures in G3, G4, and G5. Statistically significant differences between G1/G2 and G3/G4/G5.	*SBS*: G2 > G1 > G3 Differences were statistically significant. *Mode of failure*: mostly adhesive in all groups. The differences in failure mode distribution were statistically significant (*p* < 0.001). G3 exhibited a significantly greater number of mixed failures (adhesive/cohesive in build-up) and cohesive in build-up than G1 and G2. No statistically significant difference between Groups 1 and 2.	*SBS*: G2 > G3 > G1 G2 had a significantly higher SBS than G1 and G3 (*p* < 0.001). No significant differences between G3 and G1. *Mode of failure*: adhesive type was the most frequent in G2 and G3. Adhesive failure was noted in 100% of samples of G1.
Conclusions	SFC achieved SBS values comparable to those of GIC-based restorative materials routinely used to restore primary teeth.	SFC may be a reliable option to conventional materials used for the restoration of deciduous teeth especially in young or noncompliant children.	CF yielded the highest SBS to primary dentin. SFC and RMGIC demonstrated the lowest SBS with no significant difference with each other.

CF: conventional flowable composite. GIC: glass-ionomer cement. RMGIC: resin-modified glass-ionomer cement. SBS: shear bond strength. SFC: self-adhesive flowable composite.

## Data Availability

Data is available on reasonable request.
